# Analysis and Suppression of Nonlinear Error of Pendulous Integrating Gyroscopic Accelerometer at Instrument Level

**DOI:** 10.3390/s23031221

**Published:** 2023-01-20

**Authors:** Xiaojun Zhou, Gongliu Yang, Wentao Niu, Yongqiang Tu

**Affiliations:** 1 School of Instrumentation and Optoelectronic Engineering, Beihang University, Beijing 100191, China; 2 Beijing Institute of Aerospace Control Devices, Beijing 100039, China

**Keywords:** nonlinear error, pendulous integrating gyroscopic accelerometer (PIGA), error analysis and suppression, inertial navigation system (INS), instrument level

## Abstract

The error coefficients of the pendulous integrating gyroscopic accelerometer (PIGA) mainly include the bias, scale factor, and nonlinear error. Previous works have fully studied and suppressed the bias and scale factor of PIGAs. At present, the nonlinear error is the most critical factor restricting the measurement accuracy of PIGAs. To address this barrier, a study on the analysis and suppression of the nonlinear error of PIGAs at the instrument level was carried out. Firstly, the error model of a PIGA is established by kinematics and dynamics analyses. Then, nonlinear error is analyzed based on the established model. Finally, a suppression method for the nonlinear error is proposed based on the analysis results. The nonlinear error analysis found that (1) the nonlinear error includes a quadratic term error caused by unequal inertia and the inertia product, cross-coupling error is caused by lateral accelerations, and error is caused by unequal stiffness; (2) unequal inertia and the inertia product were the most critical factors resulting in nonlinear error. Based on the results in the nonlinear error analysis, the suppression method for error focuses on unequal inertia and the inertia product. The proposed method of analysis and suppression was validated experimentally as the quadratic term coefficient was reduced by an order of magnitude from 1.9 × 10^−6^/*g*_0_ to 1.91 × 10^−7^/*g*_0_.

## 1. Introduction

Accelerometers, which provide acceleration information known as the specific force of a vehicle [1], is one of the core components of an inertial navigation system (INS) [2,3]. As a dead-reckoning navigation method, the navigation accuracy of an INS is restricted to the measurement error of the accelerometer [4,5]. The most commonly used types of accelerometers for an INS include the quartz flexible accelerometer (QFA) [6,7], the microelectromechanical systems (MEMS) capacitive accelerometer [8,9], the resonant accelerometer [10,11], and the pendulous integrating gyroscopic accelerometer (PIGA) [12]. Among these accelerometer types, the PIGA is an irreplaceable high-precision inertial instrument to support INSs in submarines and ballistic vehicles owing to its advantages of much higher precision than other accelerometer types, good linearity, strong stability, and resistance to electromagnetic shock [13].

The error coefficients of the PIGA mainly include the bias irrelevant to the acceleration, the scale factor proportional to the acceleration, and the nonlinear error, including the quadratic item related to the square of the acceleration and the cross-coupling item [14,15]. The error coefficients of the bias and the scale factor of the PIGA are generally calibrated by applying inputs of ±1 $g_{0}$ ($g_{0}$ is the acceleration of gravity) on the PIGA through the positive and negative rollover tests under the conditions of a gravity field using an indexing table or a three-axis turntable [16,17,18]. In the past few decades, the focus of studies on PIGAs has been on the accuracy improvement of the bias and scale factor, and great results have been achieved as the bias stability of the PIGA can reach 0.1 µg and the scale factor stability is better than 0.1 ppm [19,20]. At present, the nonlinear error is the most critical factor restricting the measurement accuracy of the PIGA.

The nonlinear error of the PIGA can generally be calibrated by a high-acceleration calibration method using precision centrifuges, a vibrator, and a rocket sled at the instrument level [21]. In recent years, many scholars have performed studies on nonlinear error calibration for the PIGA. Wang et al. [22] analyzed the original mechanism of the cross-quadratic term and established a calibration model of the PIGA based on centrifugation to calibrate the cross-quadratic term of the PIGA. Ren et al. [23] proposed a method for calibrating the nonlinear coefficients of the PIGA on a linear vibrator by using dynamic calibration in vibrating integer periods and static calibration. Sun et al. proposed a sequential calibration method for the nonlinear errors of the PIGA on a counter-rotating platform centrifuge, which had the advantage of installing the PIGA on the centrifuge only once [24]. They also proposed a symmetric position calibration method to calibrate the main nonlinear error coefficients of the PIGA within integer precession periods on a centrifuge [25]. All these previous works have provided accurate methods for the calibration of the nonlinear error of the PIGA at the instrument level.

However, after the PIGA is installed in an INS, the error transmission mechanism at the system level is very complicated, and the nonlinear error suppression of the PIGA at the system level is not yet mature. Therefore, in the navigation and guidance model of the INS, the error coefficients of the PIGA usually only include the bias and the scale factor, and the nonlinear error is usually neglected for vehicles with short travel [26]. However, for long-distance vehicles, such as intercontinental ballistic missiles (ICBM) and ocean-going submarines that travel more than 10,000 km, small nonlinear errors will greatly reduce the precision on an INS. Taking the flight trajectory of a typical ICBM as an example, the quadratic error of the PIGA at the $1\times 10^{-5}/g_{0}$ level will cause the guidance error of the INS to exceed 200 m. Therefore, in order to improve the measurement precision of the PIGA in long-distance vehicles, it is of critical importance to analyze and suppress the nonlinear error of the PIGA at the instrument level.

In this paper, the nonlinear error of the PIGA is analyzed and suppressed at the instrument level. The remainder of the paper is organized as follows. Section 2 introduces the structure and working principle of the PIGA. Section 3 establishes the error model of the PIGA. Section 4 analyzes the nonlinear error of the PIGA based on the established error model. A suppression method for the nonlinear error is proposed in Section 5. An experimental validation for the analysis and suppression of the nonlinear error of the PIGA is presented in Section 6. The conclusions are given in Section 7.

## 2. Structure and Working Principle of PIGA

### 2.1. Structure of PIGA

**Figure 1:**
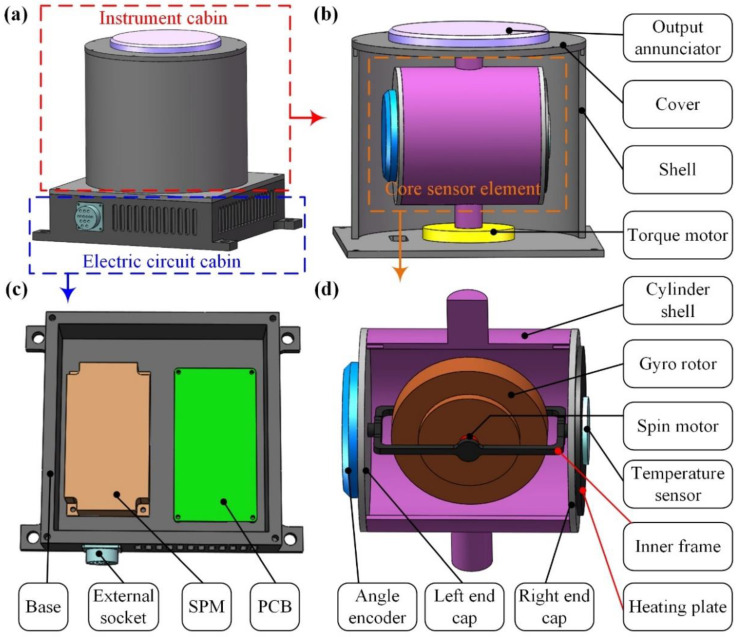
Structure of PIGA: (**a**) appearance of PIGA; (**b**) structure of the instrument cabin; (**c**) structure of the electric circuit cabin; (**d**) structure of the core sensor element.

As shown in Figure 1a, the PIGA consists of an instrument cabin and an electric circuit cabin. The components in the instrument cabin include the output annunciator, cover, shell, core sensor element, and torque motor, as shown in Figure 1b. The core sensor element is supported by the torque motor and the cover, and the rotation axis of the core sensor element coincides with the axis of the torque motor and the output annunciator. The output annunciator is used to capture the precession angular rate of the core sensor element related to the shell for the acceleration measurement. As shown in Figure 1c, the components in the electric circuit cabin include the base, external socket, secondary power module (SPM), and printed circuit board (PCB). The SPM is used to transfer external +24 V direct current (DC) to various DCs with different amplitudes to supply power to each part of the PIGA. The main functions of the PCB include: (1) capturing the output of the angle encoder by amplification/demodulation/low-pass filtering/analog-to-digital (AD) conversion, and then controlling the torque motor using proportional–integral–derivative (PID) control based on the collected signal from the angle encoder; (2) capturing the output of the temperature sensor and controlling the heating plate to maintain a constant temperature of the silicone oil in the cylinder shell; (3) capturing the output of the output annunciator and transferring the precession angular rate signal to the measured acceleration. As shown in Figure 1d, the core sensor element consists of a cylinder shell, gyro rotor, spin motor, inner frame, left end cap, angle encoder, right end cap, temperature sensor, and heating plate. The angle encoder is mounted on the left end cap and is used to measure the precession angle signal of the inner frame related to the cylinder shell. The temperature sensor and heating plate are mounted on the right end cap and are used to control the temperature of the silicone oil in the cylinder shell. The inner frame is supported by the left end cap and right end cap, and the rotation axis of the inner frame coincides with the axis of the left end cap and right end cap. The gyro rotor is supported by the inner frame, and the spin motor is used to drive the gyro rotor to rotate rapidly at a constant angular speed. The cylinder shell is filled with silicone oil to reduce the friction torque of the gyro rotor. The gyro rotor is a float gyroscope with an unbalanced pendulous mass along its pendulous axis.

With the development of the PIGA, the nonlinear error is the most critical factor restricting its measurement accuracy. However, few works have carried out research on nonlinear error analysis and suppression for the PIGA at the instrument level. The mechanism of the nonlinear error of the PIGA is not fully understood, and no suppression method has been proposed based on the error mechanism, which will largely limit the measurement accuracy after error suppression. Thus, to solve these problems, the scientific novelty of the paper is studying nonlinear error analysis and suppression for the PIGA at the instrument level for the first time. Based on this innovative point, the mechanism of the nonlinear error is fully studied, and a novel error suppression method is proposed based on the error analysis. To achieve scientific novelty, it was necessary to analyze the working principle and build a dynamic model of a PIGA as it is a mechanical inertial instrument.

### 2.2. Working Principle of PIGA

**Figure 2:**
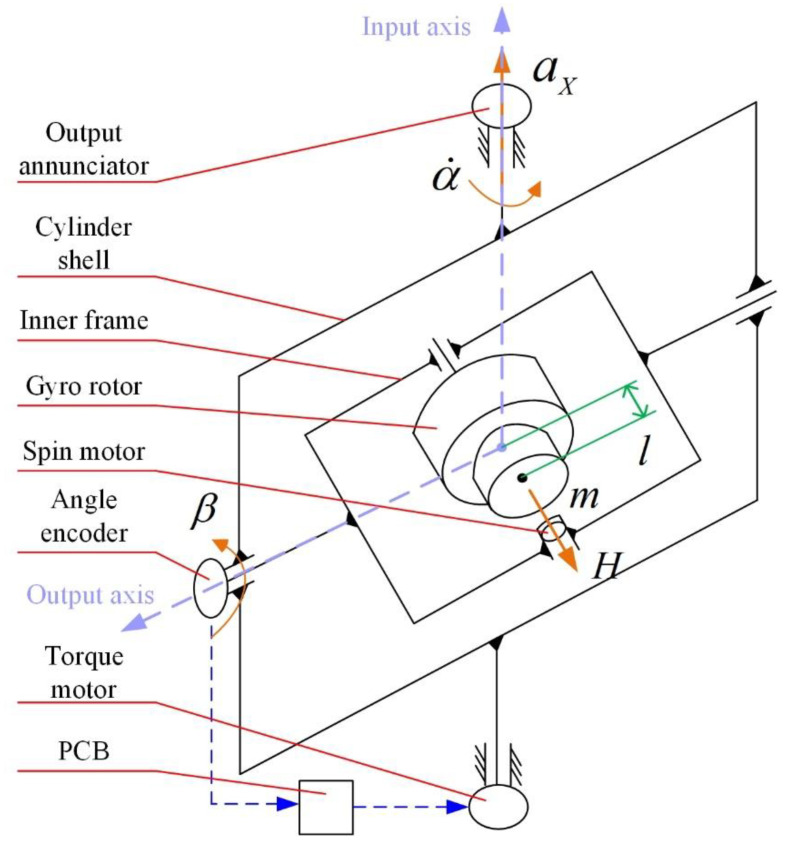
Illustration of the working principle of PIGA.

As illustrated in Figure 2, the working principle of the PIGA is as follows.

The spin motor drives the gyro rotor to rotate at a constant angular speed, and the angular momentum of the gyro rotor is H. The mass of the pendulous mass in the gyro rotor is m, and the displacement between the center of the mass of the gyro rotor and the pendulous mass is l (the center of the mass of the gyro rotor coincides with the intersection of the axes of the cylinder shell and the inner frame). The acceleration $a_{X}$ along the input axis (the input axis coincides with the axis of the cylinder shell) causes the corresponding torque $mla_{X}$ on the gyro rotor around the output axis (the output axis coincides with the axis of the inner frame) to cause precession motion of the cylinder shell around the input axis. The output annunciator is used to measure the precession angular rate $\dot{\alpha }$ of the cylinder shell.

Due to the friction torque and other disturbance torques, the inner frame will rotate around the output axis at the angle β. The angle encoder measures the β, and the PCB is used to control the torque motor based on the value of β to offset the interference torque caused by the precession of the input axis. Thus, β, $\ddot{\beta }$ ≈ 0, and the corresponding torque $mla_{X}$ is balanced with the gyro moment $H\dot{\alpha }$ as follows:

(1) $$H\dot{\alpha }=mla_{X}$$

As shown in Equation (1), the acceleration $a_{X}$ is measured by the $\dot{\alpha }$ as H and ml are both known quantities.

According to the working principle of the PIGA, it is a mechanical inertial instrument. In principle, the accuracy of the PIGA is determined by the stability of its mechanical structure as the speed of the cylinder shell’s precession indicates the magnitude of the sensitive acceleration directly. The order of magnitude of the quadratic term coefficient due to structural instability is 10^−6^/g_0_. Meanwhile, the digital measurement errors of the PIGA mainly include the measurement error of the precession angular speed of the cylinder shell using the output annunciator and the measurement error of the precession angle of the inner frame using the angle encoder. The order of magnitude of the quadratic term coefficient due to structural instability is 10^−9^/g_0_. Thus, the digital measurement errors of the PIGA can be neglected compared to the measurement error due to the structural instability. The focus of this paper is the error analysis and suppression of the nonlinear error due to the structural instability.

## 3. Error Model of PIGA

### 3.1. Kinematics and Dynamics Analyses

**Figure 3:**
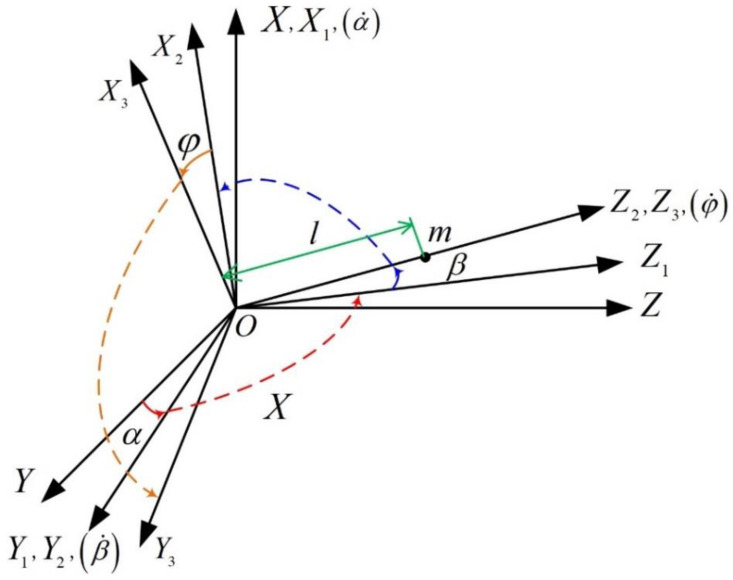
Established coordinates for kinematics and dynamics analyses of PIGA.

Firstly, the coordinates were established for the kinematics and dynamics analyses of the PIGA, as shown in Figure 3. O−XYZ is the shell coordinate fixed to the shell; $O-X_{1}Y_{1}Z_{1}$ is the cylinder shell coordinate fixed to the cylinder shell; $O-X_{2}Y_{2}Z_{2}$ is the inner frame coordinate fixed to the inner frame; $O-X_{3}Y_{3}Z_{3}$ is the gyro rotor coordinate fixed to the gyro rotor. Point O is the center of mass of the gyro rotor. Axis $OX_{1}$ coincides with the axis of the cylinder shell; axis $OY_{2}$ coincides with the axis of the inner frame; axis $OZ_{3}$ coincides with the motor axis of the spin motor. α is the precession angle of the cylinder shell around the axis $OX_{1}$; β is the rotational angle of the inner frame around the axis $OY_{2}$; φ is the rotational angle of the spin motor around the axis $OZ_{3}$.

(1) Angular velocity

It was assumed that the base has an angular velocity ω relative to the inertial space and that the projections of ω on axes OX, OY, and OZ of the coordinate O−XYZ are $\omega_{X}$, $\omega_{Y}$, and $\omega_{Z}$, respectively. According to the coordinate definitions in Figure 3, the projection of the absolute angular velocity of the cylinder shell on the coordinate $O-X_{1}Y_{1}Z_{1}$ is obtained as:

(2) $$\left[\begin{array}{c} p_{1}\\ q_{1}\\ r_{1} \end{array}\right]=\left[\left.\begin{array}{ccc} 1 & 0 & 0\\ 0 & \cos\alpha & \sin\alpha \\ 0 & -\sin\alpha & \cos\alpha \end{array}\right]\right.\left[\begin{array}{c} \omega_{X}\\ \omega_{Y}\\ \omega_{Z} \end{array}\right]+\left[\begin{array}{c} \dot{\alpha }\\ 0\\ 0 \end{array}\right]=\left[\begin{array}{c} \omega_{X}+\dot{\alpha }\\ \omega_{Y}\cos\alpha +\omega_{Z}\sin\alpha \\ -\omega_{Y}\sin\alpha +\omega_{Z}\cos\alpha \end{array}\right]$$

where $p_{1}$, $q_{1}$, and $r_{1}$ are the projections of the absolute angular velocity of the cylinder shell on axes $OX_{1}$, $OY_{1}$, and $OZ_{1}$ of the coordinate $O-X_{1}Y_{1}Z_{1}$, respectively.

The projection of the absolute angular velocity of the inner frame on the coordinate $O-X_{2}Y_{2}Z_{2}$ is:

(3) $$\begin{array}{l} \left[\begin{array}{c} p_{2}\\ q_{2}\\ r_{2} \end{array}\right]=\left[\left.\begin{array}{ccc} \cos\beta & 0 & -\sin\beta \\ 0 & 1 & 0\\ \sin\beta & 0 & \cos\beta \end{array}\right]\right.\left[\left.\begin{array}{ccc} 1 & 0 & 0\\ 0 & \cos\alpha & \sin\alpha \\ 0 & -\sin\alpha & \cos\alpha \end{array}\right]\right.\left[\begin{array}{c} \omega_{X}\\ \omega_{Y}\\ \omega_{Z} \end{array}\right]+\left[\begin{array}{c} \dot{\alpha }\cos\beta \\ \dot{\beta }\\ \dot{\alpha }\sin\beta \end{array}\right]\\ =\left[\begin{array}{c} \omega_{X}\cos\beta -\left(-\omega_{Y}\sin\alpha +\omega_{Z}\cos\alpha \right)\sin\beta +\dot{\alpha }\cos\beta \\ \omega_{Y}\cos\alpha +\omega_{Z}\sin\alpha +\dot{\beta }\\ \omega_{X}\sin\beta +\left(-\omega_{Y}\sin\alpha +\omega_{Z}\cos\alpha \right)\cos\beta +\dot{\alpha }\sin\beta \end{array}\right] \end{array}$$

where $p_{2}$, $q_{2}$, and $r_{2}$ are the projections of the absolute angular velocity of the inner frame on axes $OX_{2}$, $OY_{2}$, and $OZ_{2}$ of the coordinate $O-X_{2}Y_{2}Z_{2}$, respectively.

The projection of the absolute angular velocity of the gyro rotor on the coordinate $O-X_{2}Y_{2}Z_{2}$ is:

(4) $$\left[\begin{array}{c} p_{3}\\ q_{3}\\ r_{3} \end{array}\right]=\left[\begin{array}{c} p_{2}\\ q_{2}\\ r_{2} \end{array}\right]+\left[\begin{array}{c} 0\\ 0\\ \dot{\varphi } \end{array}\right]=\left[\begin{array}{c} \omega_{X}\cos\beta -\left(-\omega_{Y}\sin\alpha +\omega_{Z}\cos\alpha \right)\sin\beta +\dot{\alpha }\cos\beta \\ \omega_{Y}\cos\alpha +\omega_{Z}\sin\alpha +\dot{\beta }\\ \omega_{X}\sin\beta +\left(-\omega_{Y}\sin\alpha +\omega_{Z}\cos\alpha \right)\cos\beta +\dot{\alpha }\sin\beta +\dot{\varphi } \end{array}\right]$$

where $p_{3}$, $q_{3}$, and $r_{3}$ are the projections of the absolute angular velocity of the gyro rotor on axes $OX_{2}$, $OY_{2}$, and $OZ_{2}$ of the coordinate $O-X_{2}Y_{2}Z_{2}$, respectively.

(2) Angular momentum

According to the principle of the rigid body rotation dynamics [27], the angular momentums of the gyro motor and the inner frame in the coordinate $O-X_{2}Y_{2}Z_{2}$ are obtained as follows, respectively.

Under the condition that the gyro motor is fully dynamically balanced, each axis of the coordinate $O-X_{2}Y_{2}Z_{2}$ is the main axis of inertia of the motor rotor, so its inertia product is zero, and the angular momentum of the gyro rotor around the coordinate $O-X_{2}Y_{2}Z_{2}$ is written as follows:

(5) $$\left[\begin{array}{c} H_{X3}\\ H_{Y3}\\ H_{Z3} \end{array}\right]=\left[\begin{array}{c} J_{e}p_{3}\\ J_{e}q_{3}\\ J_{Z}r_{3} \end{array}\right]$$

where $H_{X3}$, $H_{Y3}$, and $H_{Z3}$ are the projections of the angular momentum of the gyro rotor on axes $OX_{2}$, $OY_{2}$, and $OZ_{2}$ of the coordinate $O-X_{2}Y_{2}Z_{2}$, respectively; $J_{e}$ and $J_{Z}$ are the radial moment of inertia and the polar moment of inertia of the gyro rotor, respectively.

In general, the axes $OX_{2}$, $OY_{2}$, and $OZ_{2}$ are not the main axes of inertia of the inner frame. Thus, its inertia product is zero, and the angular momentum of the inner frame around the coordinate $O-X_{2}Y_{2}Z_{2}$ is as follows:

(6) $$\left[\begin{array}{c} H_{X2}\\ H_{Y2}\\ H_{Z2} \end{array}\right]=\left[\begin{array}{c} J_{X2}p_{2}-J_{X2Y2}q_{2}-J_{X2Z2}r_{2}\\ -J_{X2Y2}p_{2}+J_{Y2}q_{2}-J_{Y2Z2}r_{2}\\ -J_{X2Z2}p_{2}-J_{Y2Z2}q_{2}+J_{Z2}r_{2} \end{array}\right]$$

where $H_{X2}$, $H_{Y2}$, and $H_{Z2}$ are the projections of the angular momentum of the inner frame on axes $OX_{2}$, $OY_{2}$, and $OZ_{2}$ of the coordinate $O-X_{2}Y_{2}Z_{2}$, respectively; $J_{X2}$, $J_{Y2}$, $J_{Z2}$, $J_{X2Y2}$, $J_{X2Z2}$, and $J_{Y2Z2}$ are the inertia moments and inertia products of the inner frame around the coordinate $O-X_{2}Y_{2}Z_{2}$.

(3) Dynamic equation

The combination of the gyro motor and the inner frame is defined as the float. According to Euler’s dynamic equation [28], the dynamic equation of the float relative to the inner frame axis $OY_{2}$ can be written as:

(7) $$\frac{d(H_{Y3}+H_{Y2})}{dt}+r_{2}(H_{X3}+H_{X2})-p_{2}(H_{Z3}+H_{Z2})=M_{Y2}$$

where $M_{Y2}$ is the sum of all moments acting on the inner frame axis $OY_{2}$.

Under the normal working condition of the PIGA, the value of β is stable and small. Therefore, it can be assumed that sinβ = β, cosβ = 1, $\dot{\beta }$ = 0. Equations (2)–(6) are brought into Equation (7) and Equation (7) is simplified as:

(8) $$\begin{array}{l} M_{Y2}=H\dot{\alpha }+H\left(\omega_{X}-\omega_{Z1}\beta \right)-\left[(I_{X2}-I_{Z2})\beta +J_{X2Z2}\right]\left[{\left(\dot{\alpha }+\omega_{X}\right)}^{2}-\omega_{Z1}^{2}\right]\\ +\left[(I_{Z2}-I_{X2})+4J_{X2Y2}\beta \right]\left[\left(\omega_{X}+\dot{\alpha }\right)\omega_{Z1}\right]+\left(J_{X2Y2}\beta -J_{Y2Z2}\right)\left[(\dot{\alpha }+\omega_{X})\omega_{Y1}-{\dot{\omega }}_{Z1}\right]\\ +(J_{X2Y2}+J_{Y2Z2}\beta )\left[\omega_{Y1}\omega_{Z1}+({\dot{\omega }}_{X}+\ddot{\alpha })\right] \end{array}$$

where $\left[\begin{array}{c} \omega_{X1}\\ \omega_{Y1}\\ \omega_{Z1} \end{array}\right]=\left[\left.\begin{array}{ccc} 1 & 0 & 0\\ 0 & \cos\alpha & \sin\alpha \\ 0 & -\sin\alpha & \cos\alpha \end{array}\right]\right.\left[\begin{array}{c} \omega_{X}\\ \omega_{Y}\\ \omega_{Z} \end{array}\right]=\left[\begin{array}{c} \omega_{X}\\ \omega_{Y}\cos\alpha +\omega_{Z}\sin\alpha \\ -\omega_{Y}\sin\alpha +\omega_{Z}\cos\alpha \end{array}\right]$, $I_{X2}=J_{X2}+J_{e}$, $I_{Y2}=J_{Y2}+J_{e}$, $I_{Z2}=J_{Z2}+J_{Z}$.

### 3.2. Error Model

$M_{Y2}$ in Equation (8) includes three parts: (1) the moment of inertia due to the shell acceleration; (2) the eccentric moment on axis $OY_{2}$ due to the unequal rigidity of $OX_{2}$ and $OZ_{2}$; (3) the inner frame disturbance torque, including friction torque, electromagnetic torque, elastic torque, and other disturbance torque. Thus, $M_{Y2}$ can be written as:

(9) $$M_{Y2}=-mla_{X}\cos\beta +ml(a_{Z}\cos\alpha -a_{Y}\sin\alpha )\sin\beta +M_{Y2}(B)+M_{YR}$$

where $-mla_{X}\cos\beta +ml(a_{Z}\cos\alpha -a_{Y}\sin\alpha )\sin\beta$ is the moment of inertia due to the shell acceleration; $a_{X}$, $a_{Y}$ and $a_{Z}$ are the projections of the shell acceleration on axes $OX_{2}$, $OY_{2}$ and $OZ_{2}$ of the coordinate $O-X_{2}Y_{2}Z_{2}$, respectively; $M_{Y2}(B)$ is the eccentric moment on axis $OY_{2}$ due to the unequal rigidity of $OX_{2}$ and $OZ_{2}$; $M_{YR}$ is the inner frame disturbance torque.

The PIGA is used in Platform INSs (PINSs) where $\omega_{X}$ = $\omega_{Y}$ = $\omega_{Z}$ = 0, sinβ = β, and cosβ = 1. Thus, Equation (9) is brought into Equation (8), and the error model of the PIGA is obtained as follows:

(10) $$\dot{\alpha }=K_{0}+K_{1}a_{X}+K_{2}a_{X}^{2}+\frac{ml}{H}\beta (a_{Y}\sin\alpha -a_{Z}\cos\alpha )+\frac{M_{Y2}(B)}{H}+\eta$$

where $K_{0}=\frac{M_{YR}}{H}$ is the bias irrelevant to the acceleration; $K_{1}=\frac{ml}{H}$ is the scale factor proportional to the acceleration; $K_{2}=\frac{1}{H}{\left(\frac{ml}{H}\right)}^{2}\left[(I_{X2}-I_{Z2})\beta +J_{X2Z2}\right]$ is the quadratic term coefficient related to the square of the acceleration; η is the random error of the output.

In Equation (10), the errors of $K_{0}$ and $K_{1}$ have been well studied and suppressed for the PIGA [29]. However, few studies have focused on the nonlinear error due to the quadratic term coefficient, the cross-coupling error caused by lateral acceleration, and the error caused by unequal stiffness. Therefore, to address this barrier, the focus of this study is the analysis and suppression of the nonlinear error at the instrument level.

## 4. Analysis of Nonlinear Error

According to the established error model of the PIGA (Equation (10)), the nonlinear errors includes the quadratic term error, the cross-coupling error, and the error caused by unequal stiffness.

### 4.1. Quadratic Term Error

The quadratic term error is caused by the centrifugal force generated by the inertia tensor of the float when there is precession motion of the cylinder shell around the axis $OX_{1}$. The quadratic term error is influenced by two factors: (1) the unequal inertia $(I_{X2}-I_{Z2})\beta$ and (2) the inertia product $J_{X2Z2}$.

(1) Influence of unequal inertia

**Figure 4:**
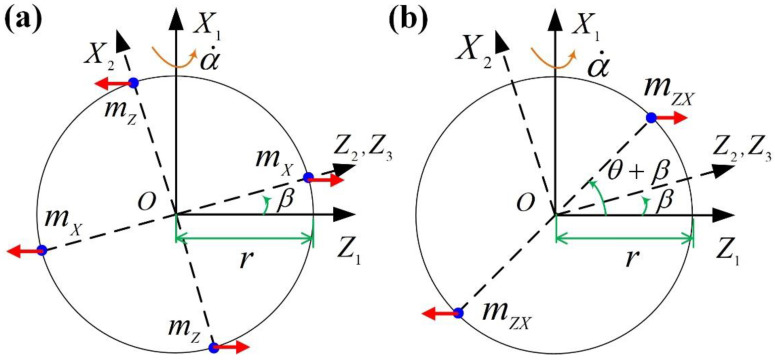
Mechanism of quadratic term error due to (**a**) unequal inertia and (**b**) inertia product.

For the PIGA, due to the existence of the pendulum mass, $I_{X2}$ is not equal to $I_{Z2}$, so the unequal inertia cannot be eliminated in principle. As illustrated in Figure 4a, $I_{X2}=2m_{X}r^{2}$; $I_{Z2}=2m_{Z}r^{2}$; when the β appears, the float does not rotate around its inertial axis, and the centrifugal force will be generated as $(I_{X2}-I_{Z2})\beta {\dot{\alpha }}^{2}$, which results in a quadratic term error due to unequal inertia.

The output error caused by centrifugal force $(I_{X2}-I_{Z2})\beta {\dot{\alpha }}^{2}$ can be expressed as $-\frac{\left[(I_{Z2}-I_{X2})\beta \right]}{H}{\left(\frac{mla_{X}}{H}\right)}^{2}$. It can be seen that the quadratic term error caused by unequal inertia is proportional to $\left(I_{X2}-I_{Z2}\right)$, β, and the square of the pendulum ${\left(ml\right)}^{2}$; it is inversely proportional to the cube of the angular momentum ${\left(H\right)}^{3}$. Under the conditions that the pendulum ml and angular momentum H of the gyro rotor are fixed and the unequal inertia cannot be reduced as the structural design requirement of the instrument, the quadratic term error caused by unequal inertia can only be suppressed by controlling the β.

(2) Influence of inertia product

The reason for the generation of the inertia product $J_{X2Z2}$ is that the mass of the float is asymmetrical along the plane $X_{2}OZ_{2}$. As illustrated in Figure 4b, $J_{X2Z2}=2m_{zx}r^{2}\sin\theta \cos\theta$. The inertia product $J_{X2Z2}$ produces a centrifugal moment along the inner frame axis for the float as $J_{X2Z2}{\dot{\alpha }}^{2}$, which results in the quadratic term error due to the inertia product.

The output error caused by the centrifugal force $J_{X2Z2}{\dot{\alpha }}^{2}$ can be expressed as $-\frac{J_{X2Z2}}{H}{\left(\frac{mla_{X}}{H}\right)}^{2}$. It can be seen that the quadratic term error caused by the inertia product is proportional to $J_{X2Z2}$ and the square of the pendulum ${\left(ml\right)}^{2}$; it is inversely proportional to the cube of the angular momentum ${\left(H\right)}^{3}$. Under the conditions that the pendulum ml and angular momentum H of the gyro rotor are fixed, $J_{X2Z2}$ should be minimized.

### 4.2. Cross-Coupling Error

According to Equation (10), the cross-coupling error is $\frac{ml}{H}\beta (a_{Y}\sin\alpha -a_{Z}\cos\alpha )$. It can be seen that due to the existence of β, the lateral accelerations ($a_{Y}$ and $a_{Z}$) are coupled to the direction of the input axis, forming the cross-coupling error. Under the action of $a_{X}$, the cylinder shell is constantly precessing relative to the shell, so the lateral accelerations relative to the cylinder shell are also constantly changing, which are periodic functions of the angle α. Therefore, it is only necessary to study the average error caused by lateral accelerations for a full circle of the cylinder shell precession.

When there is lateral acceleration $a_{Y}$, the output of the PIGA is:

(11) $$\dot{\alpha }=\frac{ml}{H}(a_{X}+a_{Y}\beta \sin\alpha )$$

Then, the time for the cylinder shell to precess one circle is:

(12) $$T=\frac{H}{ml}\int_{0}^{2\pi }\frac{d\alpha }{\left(a_{X}+a_{Y}\beta \sin\alpha \right)}=\frac{H}{mla_{X}}\frac{2\pi }{\sqrt{1-{\left(\frac{a_{Y}\beta }{a_{X}}\right)}^{2}}}$$

The influenced indicated value of the PIGA by $a_{Y}$ in one circle of the cylinder shell precession is:

(13) $$a_{Y0}=a_{X}\sqrt{1-{\left(\frac{a_{Y}\beta }{a_{X}}\right)}^{2}}=a_{X}[1-\frac{1}{2}{\left(\frac{a_{Y}\beta }{a_{X}}\right)}^{2}+\cdots ]$$

Thus, the relative error of the indicated value of the PIGA due to $a_{Y}$ is:

(14) $$\frac{a_{X}-a_{Y0}}{a_{X}}=\frac{1}{2}{\left(\frac{a_{Y}\beta }{a_{X}}\right)}^{2}$$

Similarly, the relative error of the indicated value of the PIGA due to $a_{Z}$ is:

(15) $$\frac{a_{X}-a_{Z0}}{a_{X}}=\frac{1}{2}{\left(\frac{a_{Z}\beta }{a_{X}}\right)}^{2}$$

It can be seen that the cross-coupling error is proportional to $\beta^{2}$. Since $\beta^{2}$ is very small, the cross-coupling error is also very small (the value is generally on the order of 10^-8^), which can be ignored.

### 4.3. Error Caused by Unequal Stiffness

**Figure 5:**
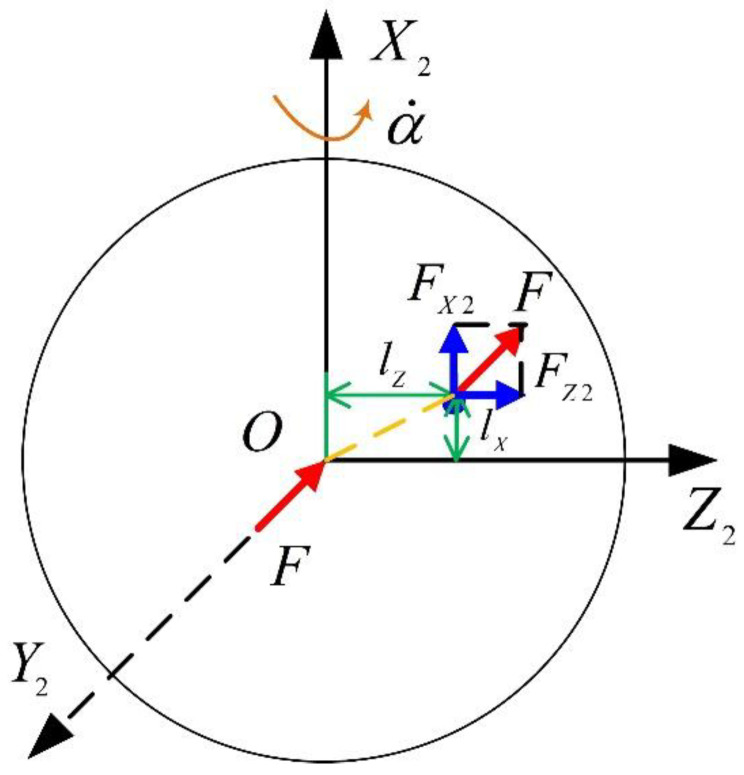
Force on the float due to unequal stiffness.

As illustrated in Figure 5, when the stiffness of the float in the direction of the cylinder shell axis and the motor axis is not equal, the acceleration $a_{X}$ will generate an additional moment on the float along the axis $OY_{2}$ as follows:

(16) $$M_{Y2}(B)=F_{Z2}l_{X}-F_{X2}l_{Z}=\frac{F_{X2}}{B_{X}}F_{Z2}-\frac{F_{Z2}}{B_{Z}}F_{X2}$$

where $l_{X}$ and $l_{Z}$ are the displacements of the center of the mass on axes $OX_{2}$ and $OY_{2}$ caused by elastic deformation, respectively; $B_{X}$ and $B_{Z}$ are the comprehensive stiffnesses along axes $OX_{2}$ and $OY_{2}$, respectively; $F_{X2}$ and $F_{Z2}$ are the forces on axes $OX_{2}$ and $OY_{2}$ caused by acceleration and can be written as:

(17) $$F_{X2}=-ma_{X}\cos\beta -(ma_{Y}\sin\alpha +ma_{Z}\cos\alpha )\sin\beta$$

(18) $$F_{Z2}=ma_{X}\sin\beta -(ma_{Y}\sin\alpha +ma_{Z}\cos\alpha )\cos\beta$$

Thus, the moment caused by the unequal stiffness is:

(19) $$M_{Y2}(B)=m^{2}[-a_{X}^{2}\beta +a_{X}(a_{Y}\sin\alpha +a_{Z}\cos\alpha )+{\left(a_{Y}\sin\alpha +a_{Z}\cos\alpha \right)}^{2}\beta ]\frac{B_{Z}-B_{X}}{B_{X}B_{Z}}$$

It can be seen that in order to reduce the error caused by unequal stiffness, it is required that the float should minimize the stiffness difference between the motor axis and the cylinder shell axis. In general, this error is very small (the value is generally on the order of 10^−9^), which can be ignored.

## 5. Suppression Method for Nonlinear Error

Based on the analysis of the nonlinear error of the PIGA in Section 4, it can be concluded that: (1) the nonlinear error includes the quadratic term error caused by unequal inertia and the inertia product, the cross-coupling error is caused by lateral accelerations, and error is caused by unequal stiffness; (2) the cross-coupling error caused by lateral accelerations and the error caused by unequal stiffness are very small, which can be ignored; (3) unequal inertia and the inertia product are the most critical factors resulting in the nonlinear error. Thus, to improve the measurement accuracy of the PIGA, suppression methods for the nonlinear error caused by unequal inertia and the inertia product are proposed in this section.

### 5.1. Suppression of Nonlinear Error Caused by Unequal Inertia

**Figure 6:**
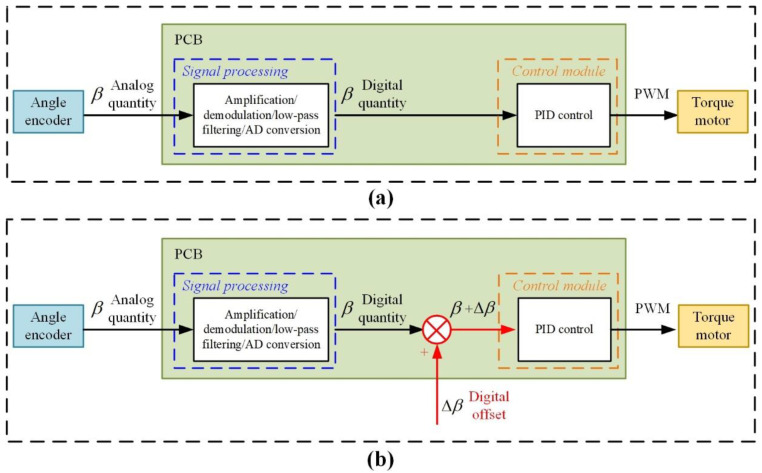
(**a**) Traditional servo control method for angle β; (**a**) proposed servo control method for angle β based on digital offset.

According to the analysis in Section 4.1, the quadratic term error caused by unequal inertia can be suppressed by controlling the β to 0. As shown in Figure 6a, the traditional servo control method for angle β is as follows: First, the angle encoder captures the analog quantity of β. Then, the analog quantities of β are processed and transferred to the digital quantities of β using modules of amplification/demodulation/low-pass filtering/AD conversion in the PCB. Afterwards, the digital quantities of β are processed and transferred into the signal of the pulse width modulation (PWM) using the control module in the PCB based on the PID control; finally, the torque motor is controlled by the signal of the PWM to counteract the disturbance torque, so that the value of β is 0.

**Figure 7:**
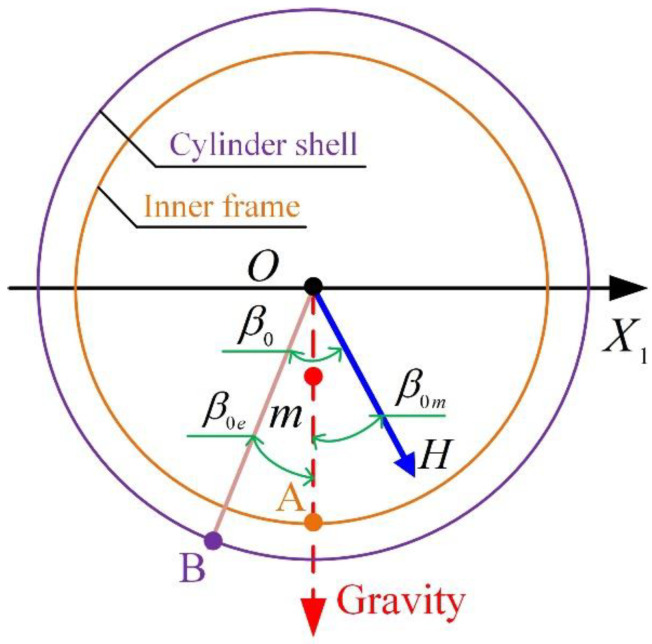
Illustration of zero offset.

However, in practice, there is a constant error of β due to the assembly error, which is defined as zero offset $\beta_{0}$. As illustrated in Figure 7, $\beta_{0}$ is produced from two assembly errors: (1) the electric assembly error $\beta_{0e}$ where point B is the zero reticle of the cylinder shell and point A is the zero reticle of the inner frame; (2) the mechanical assembly error $\beta_{0m}$. Thus, $\beta_{0}=\beta_{0e}+\beta_{0m}$.

Obviously, the traditional servo control method for angle β shown in Figure 6a can only keep the value of β at the value of $\beta_{0}$ instead of at the value of 0, which will result in nonlinear error caused by unequal inertia. To suppress the error, a novel servo control method for angle β is proposed based on the digital offset, as shown in Figure 6b. In the proposed servo control method, a signal of the digital offset Δβ is superimposed on the digital quantities of β to offset the $\beta_{0}$. The optimal Δβ was selected by the experimental method: First, Δβ is set from −5 s to 5 s in intervals of 0.5 s as $\Delta \beta_{i}$ (i = 1,2,…,21). The reason for choosing 0.5 s as the interval of setting the digital offset is that the order of magnitude of the quadratic term coefficient due to the 0.5 s digital offset near the zero position is 10^−8^/g_0_, which is an order of magnitude higher than the calibration accuracy of *K_2_*. Then, for each $\Delta \beta_{i}$, the PIGA is mounted on the counter-rotating platform centrifuge and the quadratic term coefficient $K_{2}$ is calibrated using the method in [26]. The calibration accuracy of *K_2_* using precision centrifuge is 10^−7^/g_0_. Finally, to guarantee that a global minimum is obtained, the optimal Δβ is selected for minimum value of $K_{2}$ using a two-step experimental data processing method: (1) Step 1: The fitted curve of $K_{2}$-Δβ is obtained using spline interpolation in intervals of 0.001 s based on the experimental data, as spline interpolation is more powerful and flexible than the classical methods based on algebraic and trigonometric polynomials [30]. (2) Step 2: The line-search method is used to find the best Δβ for the minimum value of $K_{2}$ based on the fitted curve.

### 5.2. Suppression of Nonlinear Error Caused by Inertia Product

A method based on the float inertia product correction is proposed to suppress the nonlinear error caused by the inertia product. According to Equation (10), the inertia product $J_{X2Z2}$ can be calculated using the calibrated $K_{2}$ after β is offset using the proposed servo control method in Section 5.1, as follows:

(20) $$J_{X2Z2}=K_{2}H/{\left(\frac{ml}{H}\right)}^{2}$$

After $J_{X2Z2}$ is calculated, the float is removed from the PIGA and $J_{X2Z2}$ is adjusted using a laser fine-weight adjusting machine (LFWAM) by removing the mass. Finally, the float is installed in the PIGA again to reduce the nonlinear error caused by $J_{X2Z2}$. 

**Figure 8:**
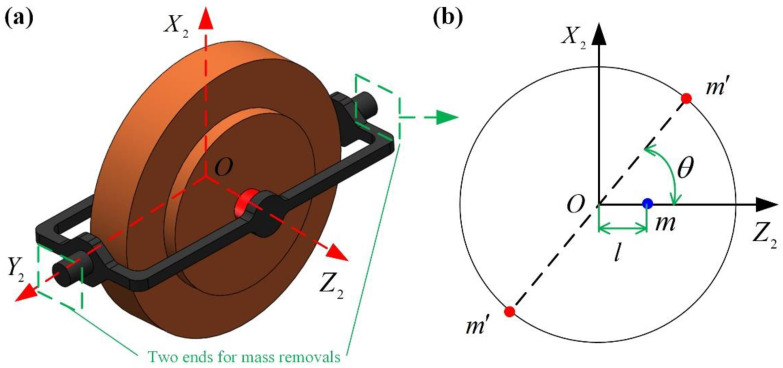
Illustration of the mass removal: (**a**) two ends for mass removal; (**b**) details of mass removal on each end.

In order to minimize the impact of the removed mass on the pendulum, the liquid floating balance and the working temperature of the float, two points of mass removal, are determined at each end of the inner frame along axis $OY_{2}$ as shown in the Figure 8a.

As shown in Figure 8b, the adjusted $J_{X2Z2}$ by mass removal is:

(21) $$J_{X2Z2}=2\times 2m’b^{2}\sin\theta \cos\theta =2m’b^{2}\sin2\theta$$

where m’ is the removed mass; b is the distance between the center of the gyro rotor and the position of the mass removal; θ is the angle between the removed mass and the pendulous mass.

To minimize the m’, θ is set as 45° and b is set as $r_{f}$ ($r_{f}$ is the radius of the end surface). Thus, the removed mass m’ is calculated as follows:

(22) $$m’=\frac{1}{2}J_{X2Z2}/r_{f}^{2}=\frac{m^{2}l^{2}}{2Hr_{f}^{2}}K_{2}$$

## 6. Experimental Validation and Discussion

**Figure 9:**
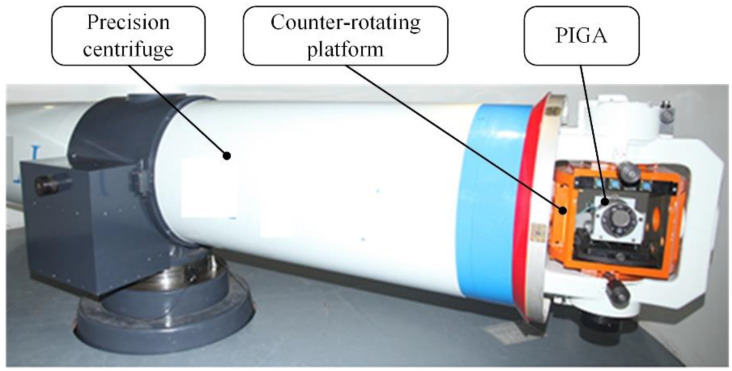
Experimental setup for calibration of $K_{2}$.

**Figure 10:**
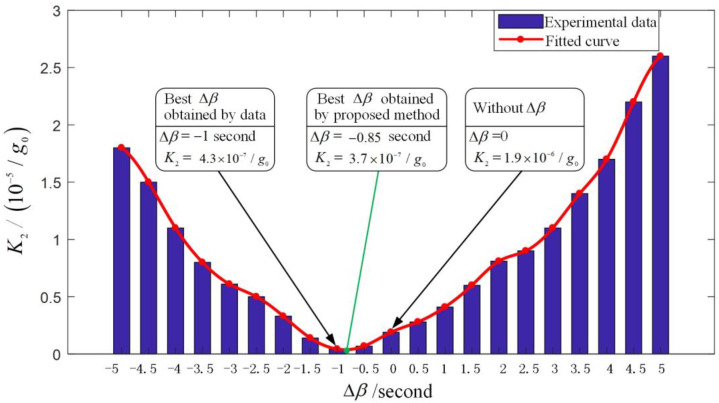
Measured quadratic term error under each $\Delta \beta_{i}$ (i = 1, 2,…,21) and best Δβ selection using the proposed method.

In order to validate the proposed method of analysis and suppression of the nonlinear error of the PIGA, a PIGA was used to conduct error suppression based on the proposed method. Firstly, the suppression of the nonlinear error caused by unequal inertia was conducted. The experimental setup for the calibration of $K_{2}$ is presented in Figure 9. The $K_{2}$ under each $\Delta \beta_{i}$ (i = 1, 2,…,21) is plotted in Figure 10. As shown in Figure 10, the value of $K_{2}$ was $1.9\times 10^{-6}/g_{0}$ without Δβ. The best Δβ was selected as −1 s using the experimental data directly, as $K_{2}$ was at the minimum value of $4.3\times 10^{-7}/g_{0}$ among the experimental data when the interval of Δβ was 0.5 s. The best Δβ was selected as −0.85 s using spline interpolation and line search, as $K_{2}$ was at the minimum value of $3.7\times 10^{-7}/g_{0}$ among the fitted data when the interval of Δβ was 0.001 s. Thus, the proposed method of suppression of the nonlinear error caused by unequal inertia was validated, as $K_{2}$ was reduced from $1.9\times 10^{-6}/g_{0}$ to $3.7\times 10^{-7}/g_{0}$ and the value of the obtained $K_{2}$ is also less than the minimum value among the experimental data.

After the suppression of the nonlinear error caused by unequal inertia, the suppression of the nonlinear error caused by the inertia product was conducted. The removed mass was calculated as 77.8 mg using Equation (22), and mass removal was conducted using the LFWAM. Finally, after the suppression, a counter-rotating platform centrifuge was used to calibrate the $K_{2}$, and the result was $1.91\times 10^{-7}/g_{0}$. Thus, the proposed method of suppression of the nonlinear error of the PIGA was validated as the $K_{2}$ was reduced by an order of magnitude from $1.9\times 10^{-6}/g_{0}$ to $1.91\times 10^{-7}/g_{0}$.

In this paper, the mechanism of the nonlinear error is fully studied and a novel and effective error suppression method is proposed based on the error analysis. Compared to state-of-the-art studies in related fields (for example, Belkhouche [31] proposed a mathematical model for calibration accelerometer systems in 2018; Ren et al. [23] proposed an approach for calibrating the nonlinear coefficients of the PIGA; Sun et al. [24] developed a sequential calibration method for the nonlinear errors of the PIGA on a counter-rotating platform centrifuge), the advantages of the proposed method are: (1) the nonlinear errors are analyzed based on the working principle and dynamic modeling at the instrument level, which can help researchers to fully understand the errors; (2) the suppression method is based on the nonlinear error analysis, so the measurement accuracy after the suppression is much higher than the existing calibration-based error compensation methods.

## 7. Conclusions

In this paper, a study on the analysis and suppression of the nonlinear error of the PIGA at the instrument level is presented. First, the structure and working principle of the PIGA were introduced. Then, an error model of the PIGA was established by kinematics and dynamics analyses. Afterwards, the nonlinear error was analyzed based on the established model. Then, a suppression method for nonlinear error was proposed based on the analysis results. Finally, experiments were conducted to validate the proposed method. For the nonlinear error analysis, it was found that (1) the nonlinear error includes the quadratic term error caused by unequal inertia and the inertia product, the cross-coupling error is caused by lateral accelerations, and error is caused by unequal stiffness; (2) the cross-coupling error caused by lateral accelerations and the error caused by unequal stiffness are very small and can be ignored; (3) unequal inertia and the inertia product are the most critical factors resulting in the nonlinear error. Thus, to improve the measurement accuracy of the PIGA, suppression methods for the nonlinear error caused by unequal inertia and the inertia product are proposed. For the suppression of the nonlinear error caused by unequal inertia, a novel servo control method for the angle β is proposed based on the digital offset. For the suppression of the nonlinear error caused by the inertia product, a method for $J_{X2Z2}$ adjustment based on mass removal using a laser-fine weight-adjusting machine (LFWAM) is proposed. The proposed method of analysis and suppression of the nonlinear error of the PIGA was validated experimentally, as the quadratic term coefficient was reduced by an order of magnitude from $1.9\times 10^{-6}/g_{0}$ to $1.91\times 10^{-7}/g_{0}$.

In future work, we will conduct research on nonlinear error analysis and suppression of the PIGA at the system level.

## Data Availability

Not applicable.
